# Establishment and validation of a risk prediction model for delayed neurocognitive recovery associated with cerebral oxygen saturation monitoring

**DOI:** 10.1186/s13741-024-00432-5

**Published:** 2024-07-16

**Authors:** Ning Luo, Xiaowei Gao, Chunyan Ye, Lu Wang, Lu Tang, Yongqiu Xie, E. Wang

**Affiliations:** 1 Department of Anesthesiology, Xiangya Hospital Central South University, Changsha, 410008 China; 2 National Clinical Research Center for Geriatric Disorders (Xiangya Hospital), Xiangya Road #87, Changsha, Hunan 410008 China

**Keywords:** Delayed neurocognitive recovery, Laparoscopic surgery, Predictive model, Cerebral oxygen saturation, Postoperative

## Abstract

**Background:**

Delayed neurocognitive recovery (DNR) is a common complication in patients undergoing laparoscopic surgery, and there are currently no effective therapies. It is vital to provide a reliable basis for clinical prediction. This study tried to analyse the risk factors for DNR in patients undergoing laparoscopic colorectal surgery and to establish a risk prediction model.

**Methods:**

A retrospective analysis of the clinical data and DNR status of patients undergoing laparoscopic colorectal surgery at Xiangya Hospital of Central South University from March 2018 to July 2020 was conducted. Logistic regression was performed to analyse the related risk factors for DNR post-operatively, and the predictive model of DNR post-operatively was constructed and validated internally. Patients who underwent laparoscopic colorectal surgery between January and July 2021 were also selected for external validation of the predictive model, to ultimately investigate the risk factors for DNR in patients undergoing laparoscopic colorectal surgery.

**Results:**

The incidence of DNR in patients undergoing laparoscopic colorectal surgery was 15.2% (31/204). The maximum variability of cerebral oxygen, age, education, and pre-existing diabetes was related to the incidence of DNR (*p* < 0.05). The risk prediction model of DNR after laparoscopic colorectal surgery was established. The internal and external validation showed that the discrimination was good (the AUCs were 0.751 and 0.694, respectively).

**Conclusions:**

The risk prediction model of DNR related to cerebral oxygen saturation monitoring shows good predictive performance and clinical value, providing a basis for postoperative DNR prevention.

## Background

Laparoscopic surgery is a standard treatment for colorectal cancer which is one of the most common malignant tumours, ranking 4th in morbidity and mortality (Bray et al. 2018). However, a long duration of intraoperative CO_2_ pneumoperitoneum has been shown to increase PaCO_2_, intraperitoneal pressure, and excitation of the sympathetic-adrenal medulla system. This pathophysiology increases the incidence of postoperative surgical complications, including cognitive dysfunction (POCD) (Ding et al. 2018). POCD is a disorder of the thinking process of patients that occurs after anaesthesia and surgery. The symptoms may be transient or include severe memory impairment, psychomotor impairment, and dementia (Lu et al. 2020; Phillips-Bute et al. 2006), which leads to the extension of hospitalization, an increase in expenses, and a potentially decreased long-term quality of life of patients, imposing a heavy burden on the family and society (Boone et al. 2020; Steinmetz et al. 2009). Delayed neurocognitive recovery (DNR) is a subtype of POCD (Steinmetz and Rasmussen 2016), and there are currently no effective therapies. Therefore, it is vital to provide a reliable basis for clinical prediction.

In this study, the perioperative clinical data of patients undergoing laparoscopic colorectal cancer surgery were collected retrospectively, and the aim was to discover the potential risk factors and to establish a predictive model of DNR to provide a reference for the clinical prediction of the possibility of DNR in postoperative patients.

## Methods

### Subjects and cohort

A total of 234 eligible patients from Xiangya Hospital of Central South University from March 2018 to July 2021 were included. The eligibility criteria were as follows: (1) age ≥ 50; (2) American Association of Anaesthesiologists (ASA) Classification II–III; (3) patients undergoing selective laparoscopic colorectal cancer surgery under general anaesthesia; and (4) expected postoperative hospitalization of 5 days or more. The exclusion criteria were as follows: (1) patients undergoing emergency surgery or in a coma; (2) inability to communicate due to language, hearing, or vision impairment; (3) preoperative history of stroke, transient ischemia attack (TIA), schizophrenia, Parkinson’s disease, epilepsy, or dementia; (4) severe heart disease, including preoperative left ventricular ejection fraction less than 30% or arrhythmias with pacemaker placement; (5) requirement for liver transplantation or liver dysfunction with Child–Pugh grade C; (6) preoperative renal dysfunction requiring dialysis; (7) expected survival time of less than 24 h; and (8) diagnosed with emergence delirium (ED) or postoperative delirium (POD). The patients were divided into the DNR group or the control group based on whether DNR occurred after the operation.

A total of 204 patients for whom data were collected between March 2018 and July 2019 were included in the modelling group for the predictive model. Clinical data for 25 patients collected between January and July 2021 were used for external validation of the predictive model.

### Design and setting

Patients were induced with etomidate 0.3–0.4 mg/kg, sufentanil 0.5 μg/kg, and cisatracurium 0.1–0.15 mg/kg. Anaesthesia was maintained with a target-controlled infusion of propofol and remifentanil. Intermittent intravenous infusion of rocuronium/cisatracurium was performed to maintain muscle relaxation. The heart rate and blood pressure were maintained within 20% of the baseline during the operation. All incisions after suturing were treated with 1% ropivacaine local infiltration analgesia.

The general, clinical, and perioperative data were collected, which included age, gender, body mass index (BMI), years of education, preoperative diagnosis, preoperative laboratory examination, operative methods, anaesthetic methods, duration of surgery, intra-abdominal pressure (IAP), intraoperative medication, intraoperative positioning, volume of transfusion, volume of blood transfusion, volume of haemorrhage, volume of urine, and cerebral oxygen saturation.

The patients were administered neuropsychological tests: Confusion Assessment Method Intensive Care Unit (CAM-ICU), Richmond Agitation-Sedation Scale (RASS), and the Mini-mental State Examination (MMSE) scores were recorded 1 day pre-operatively and 7 days post-operatively by specifically trained staff. With the MMSE score on the day before surgery as the baseline score, a diagnosis of DNR is made if the MMSE score on the 7th day after surgery decreases by 3 points or more compared to the baseline score (Chen et al. 2022; Wang et al. 2021). The study was in compliance with the Strengthening the Reporting of Observational Studies in Epidemiology (STROBE) reporting guidelines.

### Statistical analysis

Analysis was performed using SPSS 24.0 statistical software (SPSS, Inc., Chicago, IL). Use SPSS to perform a normality test on all numeric variables, including age, BMI, years of education, preoperative haemoglobin concentration, intraoperative blood loss, duration of surgery, IAP, baseline rSO_2_, and maximum variability of rSO_2_. The variables that exhibited a normal distribution are described by the mean ± standard deviation (SD), and the variables that were not normally distributed are described by the median and interquartile distance (IQR). The classification variables are described by numbers and percentages (%). A two-sample *t*-test was used for the comparison between the groups of measurement data conforming to a normal distribution, and the Mann–Whitney U test was used for the comparison between the groups of measurement data conforming to a non-normal distribution. Enumeration data were compared by the *χ*^2^ test. A logistic regression model was used to screen the significant factors to construct the risk prediction model, and a receiver operating characteristic curve was used to analyse the prediction efficiency of the model. The Hosmer–Lemeshow goodness-of-fit test and calibration curves were used to evaluate the fitting degree of the prediction model. Furthermore, the RMS program package of R language (version 4.1.2) was used to establish the risk profile of postoperative DNR in colorectal cancer patients. Then, the bootstrap method was used to repeat sampling 1000 times for internal validation. External validation of the model was conducted using data collected between January and July 2021. All statistical analyses revealing differences of *p* < 0.05 were statistically significant.

## Results

### Comparison of clinical data between the modelling and validation groups

There were no significant differences in gender, BMI, years of education, blood loss, rate of comorbidities (e.g. hypertension, coronary heart disease, and diabetes mellitus), or maximum variability of rSO_2_ between the two groups (*p* > 0.05). The validation group exhibited a higher average age, increased incidence of DNR, and a greater frequency of head-down positioning. In contrast, their preoperative haemoglobin concentration, surgery duration, and intra-abdominal pressure were lower compared to the modelling group. There was a significant difference between the two groups (*p* < 0.05, Table 1).

**Table 1:** Demographics of patients undergoing laparoscopic colorectal cancer surgery in the training and validation cohorts

	Modelling group (*n* = 204)	Validation group (*n* = 25)	*P* value
Age, mean ± SD, year	62.99 ± 7.49	68.40 ± 4.90	0.015
Gender			0.123
Male, *n* (%)	133 (65.2%)	12 (48.0%)	
Female, *n* (%)	71 (34.8%)	13 (52.0%)	
BMI, mean ± SD, kg/m^2^	22.82 ± 2.94	23.46 ± 3.62	0.650
Years of Education, mean ± SD, year	9.09 ± 3.78	8.84 ± 3.80	0.941
Preop Hb, median (IQR), g/L	128 (115, 138)	112 (100.5, 127.5)	0.005
Intraop blood loss, median (IQR), mL	100 (50, 150)	100 (50, 175)	0.400
Duration of surg, mean ± SD, min	187.54 ± 79.89	177.80 ± 46.95	0.020
Intraoperative positioning			< 0.001
Head down, *n* (%)	115 (56.4%)	21 (84.0%)	
Head up, *n* (%)	9 (4.4%)	4 (16.0%)	
Supine position, *n* (%)	73 (35.8%)	0 (0%)	
IAP, median (IQR), mmHg	15 (14, 15)	12.5 (12, 13)	< 0.001
Baseline rrSO_2_, mean ± SD, %	64.39 ± 5.96	65.74 ± 5.05	0.426
Baseline lrSO_2_, mean ± SD, %	64.54 ± 5.35	63.80 ± 3.83	0.034
lrSO_2_ v, mean ± SD, %	11.14 ± 8.13	8.17 ± 6.08	0.480
rrSO_2_ v, mean ± SD, %	13.45 ± 8.57	9.91 ± 6.53	0.167
Diabetes			0.756
No, *n* (%)	177 (86.8%)	21 (84.0%)	
Yes, *n* (%)	27 (13.2%)	4 (16.0%)	
Hypertension			0.481
No, *n* (%)	144 (70.6%)	20 (80.0%)	
Yes, *n* (%)	60 (29.4%)	5 (20.0%)	
CAD			1.000
No, *n* (%)	192 (94.1%)	24 (96.0%)	
Yes, *n* (%)	12 (5.9%)	1 (4.0%)	
DNR			0.021
No, *n* (%)	173 (84.8%)	16 (64.0%)	
Yes, *n* (%)	31 (15.2%)	9 (36.0%)	

The values are presented as means ± SD (standard deviation), median (interquartile range), or *n* (%) depending on type and distribution

*IQR* interquartile range, *BMI* body mass index, *Preop Hb* preoperative haemoglobin concentration, *IAP* intra-abdominal pressure, *rSO2v* the maximum variability of cerebral oxygen, *CAD* coronary artery disease, *DNR* delayed neurocognitive recovery

### Establishment of a risk prediction model for DNR after laparoscopic surgery for colorectal cancer

#### General conditions of patients in the modelling group

Of the 204 patients in the modelling group, 31(15.2%) showed delayed neurocognitive recovery. There were no significant differences between the two groups in terms of gender, age, BMI, years of education, preoperative haemoglobin concentration, intra-abdominal pressure, surgery duration, intraoperative positioning, percentage of pre-existing hypertension, coronary heart disease, or baseline rSO_2_ (*p* > 0.05). However, the maximum variability of left rSO_2_ was higher in the DNR group (*p* < 0.001). Compared to the control group, patients in the DNR group experienced greater intraoperative blood loss and had a higher prevalence of pre-existing diabetes. The differences were statistically significant (*p* < 0.05, Table 2).

**Table 2:** Clinical data of patients in the DNR and control groups

	DNR group (*n* = 31)	Control group (*n* = 173)	*P* value
Age, mean ± SD, year	66.32 ± 7.90	62.39 ± 7.28	0.755
Gender			0.153
Male, *n* (%)	24 (77.4%)	109 (63.0%)	
Female, *n* (%)	7 (22.6%)	64 (37.0%)	
BMI, mean ± SD, kg/m^2^	22.75 ± 2.41	22.83 ± 3.03	0.108
Years of education, mean ± SD, year	8.03 ± 3.75	9.28 ± 3.77	0.715
Preop Hb, median (IQR), g/L	128 (121, 136)	129 (113.5, 139.0)	0.731
Intraop blood loss, median (IQR), mL	100 (100, 200)	100 (50, 150)	0.040
Duration of surg, mean ± SD, min	212.03 ± 77.15	183.15 ± 79.79	0.666
IAP, median (IQR), mmHg	14 (14, 15)	15(14, 15)	0.162
Intraoperative positioning			0.423
Head down, *n* (%)	3 (9.7%)	6 (3.5%)	
Head up, *n* (%)	15 (48.4%)	100 (57.8%)	
Supine position, *n* (%)	12 (38.7)	61 (35.3%)	
Baseline rrSO_2_, mean ± SD, %	63.35 ± 5.96	64.57 ± 5.96	0.976
Baseline lrSO_2_, mean ± SD, %	62.39 ± 5.55	64.93 ± 5.23	0.914
lrSO_2_ v, mean ± SD, %	13.61 ± 13.44	10.70 ± 6.73	< 0.001
rrSO_2_ v, mean ± SD, %	13.33 ± 9.15	13.47 ± 8.49	0.372
Diabetes, *n* (%)	8 (25.8%)	19 (11.0%)	0.040
Hypertension, *n* (%)	8 (25.8%)	52 (30.1%)	0.831
CAD, *n* (%)	2 (6.5%)	10 (5.8%)	1.000

The values are presented as means ± SD (standard deviation), median (interquartile range), or n (%) depending on type and distribution

*DNR* delayed neurocognitive recovery, *IQR* interquartile range, *BMI* body mass index, *Preop Hb* preoperative haemoglobin concentration, *IAP* intra-abdominal pressure, *rSO2v* the maximum variability of cerebral oxygen, *CAD* coronary artery disease

#### Screening risk factors for DNR by multiple logistic regression analysis

The logistic regression equation, which included age, years of education, the maximum variability of left rSO_2_, volume of intraoperative blood loss, and rate of comorbidities (e.g. hypertension, coronary heart disease, and diabetes mellitus), was constructed. The results indicated that the risk of DNR increased with age (*p* = 0.007), decreased with an increase in years of education (*p* = 0.037), and increased with the presence of diabetes mellitus (*p* = 0.009). Furthermore, an increase in the maximum variability of left rSO_2_ also elevated the risk of DNR (*p* = 0.035) (Table 3).

**Table 3:** Multivariate logistic regression analysis of factors affecting DNR in patients undergoing laparoscopic colorectal cancer surgery

	β	SE	Wald *χ*^2^	*P*	OR	95% CI of OR
lrSO_2_v	0.047	0.022	4.458	0.035	1.048	1.003 ~ 1.095
Age	0.085	0.031	7.335	0.007	1.088	1.024 ~ 1.157
Years of education	− 0.122	0.059	4.345	0.037	0.885	0.789 ~ 0.993
Intraop blood loss	0.001	0.001	0.438	0.508	1.001	0.999 ~ 1.003
Diabetes	1.492	0.573	6.780	0.009	4.448	1.446 ~ 13.679
Hypertension	− 0.727	0.522	1.943	0.163	0.483	0.174 ~ 1.34
CAD	− 0.401	0.914	0.193	0.661	0.670	0.112 ~ 4.016
Constant	− 6.825	2.205	9.581	0.002	0.001	

*DNR* delayed neurocognitive recovery, *rSO2v* the maximum variability of cerebral oxygen, *OR* odds ratio, *CAD* coronary artery disease

#### Establishment of risk prediction model of DNR

**Fig. 1:**
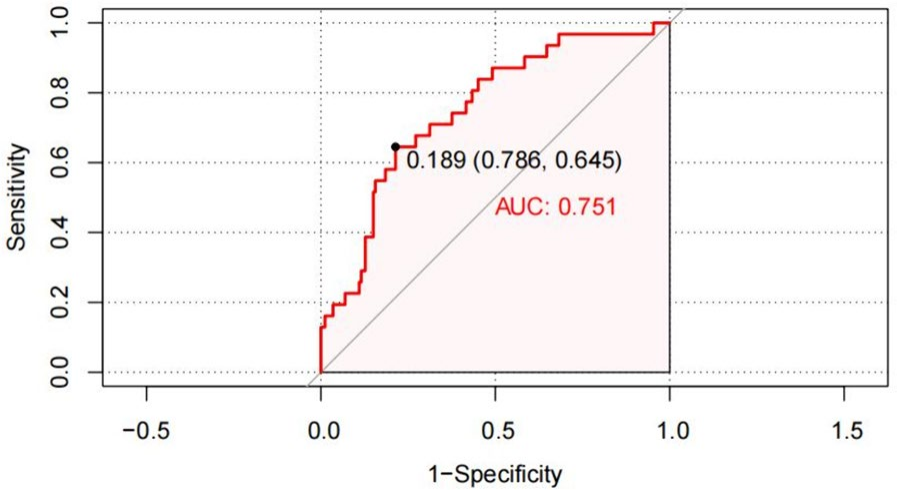
ROC curve of the model for DNR in patients undergoing laparoscopic surgery for colorectal cancer

Based on the results of the multivariate regression analysis, a predictive model was established: Logit (*p*) =  − 6.825 + 0.047 × lrSO_2V_ − 0.122 × years of education + 0.085 × age + 1.492 × with diabetes. The ROC curve of the model was plotted (Fig. 1). The area under the curve (AUC) was 0.751 (95% CI, 0.661–0.842, *p* < 0.001), and cut-off value was 0.189, which showed that the discrimination was good.

**Fig. 2:**
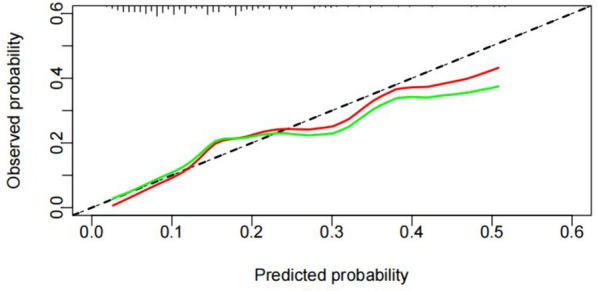
Calibration curve of the model for DNR in patients undergoing laparoscopic surgery for colorectal cancer

The calibration degree of the model was tested by the Hosmer–Lemeshow goodness-of-fit test, and a calibration curve was generated. There was no significant difference between the predicted and observed values (Hosmer–Lemeshow *χ*^2^ = 9.460, *p* = 0.305). At the same time, the calibration curve and the prediction curve fit well, which indicated that the model had a good fitting degree and a high calibration degree (Fig. 2).

#### Establishment of the risk profile nomogram for DNR after laparoscopic colorectal cancer surgery

**Fig. 3:**
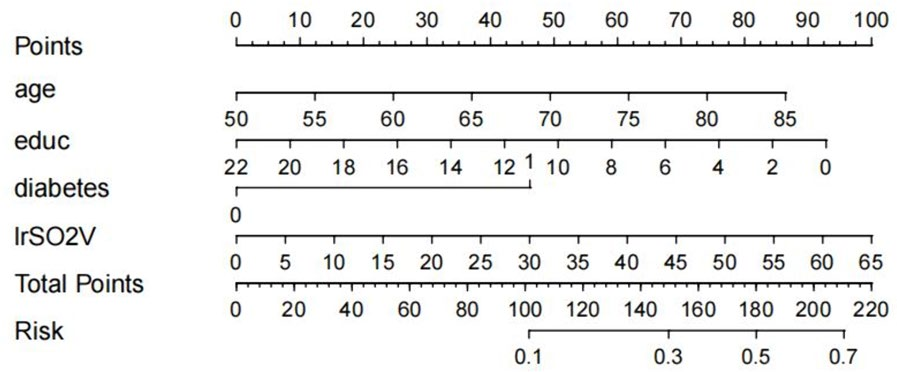
A nomogram for predicting risks of DNR in patients undergoing laparoscopic surgery for colorectal cancer

Based on the results of the multivariate analysis above, the risk profile of DNR was further established using R Studio software (Fig. 3). Suppose an 80-year-old patient with colorectal cancer, a high school education, preoperative diabetes, and intraoperative monitoring of left rSO_2_ with a maximum variability of 15% has a total score of 74 + 41 + 45 + 23 = 183. The risk of postoperative DNR is approximately 52%. According to the nomogram, clinicians can easily, visually, and accurately assess the risk of postoperative DNR and formulate individualized prevention and intervention measures.

### Validation of the risk prediction model for DNR after laparoscopic colorectal cancer surgery

**Fig. 4:**
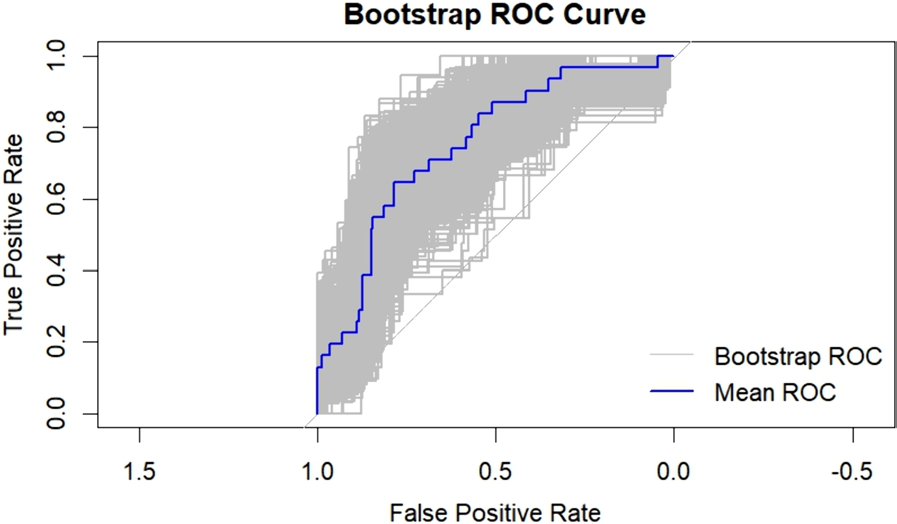
ROC curve of the risk prediction model for the internal validation using the Bootstrap method

Internal validation: The AUC of the model is 0.751 (95% CI, 0.661–0.842), which is calculated by using the bootstrap method and repeated sampling 1000 times (Fig. 4).

**Fig. 5:**
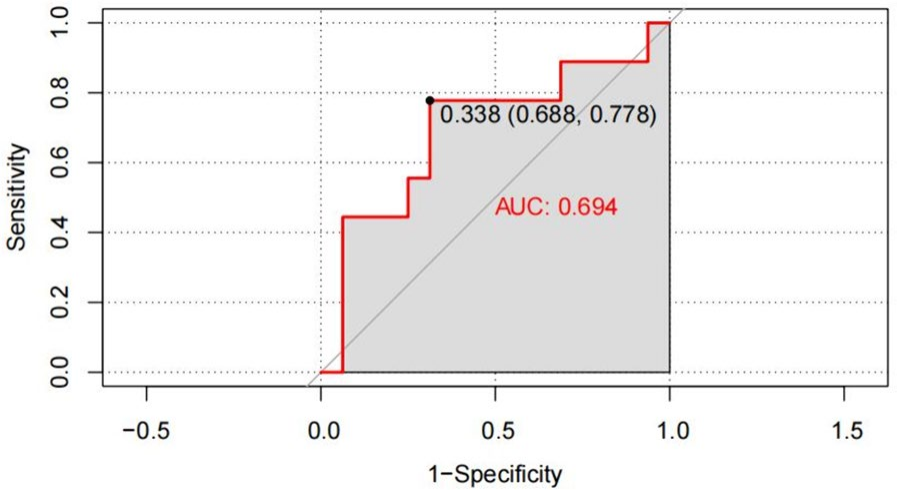
ROC curve of the risk prediction model for DNR in the validation group

External validation: The C index was calculated to be 0.694 (95% CI, 0.385–0.879) using the data collected from January to July 2021, and the Hosmer–Lemeshow goodness-of-fit test results (*χ*^2^ = 11.631, *p* = 0.1684) suggested that the prediction model had moderately good discrimination and fit for the external data (Fig. 5).

## Discussion

Delayed neurocognitive recovery (DNR) is a new term proposed in 2018 in the same article published in several anaesthesiology-related journals. It refers to the period from the end of surgery to 30 days after surgery, with the exception of POD and cognitive decline in patients, renamed postoperative cognitive dysfunction (POCD) (Evered et al. 2018). In previous studies, the incidence of DNR varied widely, with approximately 50–70% of hospitalized patients undergoing cardiac surgery (Newman et al. 2006; Selnes et al. 1999). Inouy et al. reported a 13–50% incidence of DNR in elderly patients undergoing noncardiac surgery (Inouye et al. 2014). In this study, the incidence of DNR was 15.2% among patients undergoing laparoscopic surgery for colorectal cancer. In the reported literature, the long-term mortality of the POCD group was higher than that of the age- and sex-matched control group without POCD. Therefore, screening out meaningful risk factors and constructing risk prediction models are helpful for clinicians to take early intervention measures according to patients’ conditions to prevent DNR and improve patients’ long-term prognosis.

Ageing is the only known risk factor for DNR. With increasing age, brain volume declines, and changes in the central nervous system occur, which in turn affect cognitive function (Moller et al. 1998; Monk et al. 2008). In addition, the age-related dysfunction of the blood–brain barrier caused by surgery with anaesthesia found by Yang et al. (2017), and the decrease in cerebral blood flow caused by a decrease in masticatory function found by Luo et al. could be significant causes of DNR in elderly patients (Yang et al. 2017). In this study, the risk of developing DNR increased to 1.088 times in older patients.

This study shows that long-term education is a protective factor for DNR. In a previous study conducted by Buanes et al. (Yang et al. 2017), the incidence of postoperative cognitive dysfunction was lower in those with higher education than in those with lower education, which is consistent with the results of this study. This may be because brain activity increases in higher-educated older adults, which compensates for pathological changes in the brain after anaesthetized surgery by storing more neurons, bypassing damaged areas, and increasing synaptic efficiency, leading to attenuation of the effects of anaesthesia on cognitive function.

Diabetes mellitus was an independent risk factor for DNR in this study. I. Feinkohl’s meta-analysis revealed that people with diabetes were 1.26 times more likely to develop postoperative cognitive dysfunction (Feinkohl et al. 2017). The main reason may be that glucose-induced advanced glycation end-products react with specific cell surface receptors, resulting in neurodegeneration and arteriosclerosis, while the harm of anaesthesia exacerbates the vulnerability of diabetic patients to such neuropathological changes, leading to a greater impact of anaesthesia on cognitive function than in nondiabetic patients (Brownlee 2001). In this study, the risk of postoperative DNR was 4.448-fold higher in patients with diabetes than in patients without diabetes.

This study showed that an increase in the maximum variability of intraoperative cerebral oxygen saturation (rSO_2_) value is a risk factor for DNR. Recent studies on the relationship between rSO_2_ and POCD have yielded discrepant results; many studies have confirmed that the degree of rSO_2_ decline is a risk factor for POCD (Kim et al. 2016; Lin et al. 2013; Nielsen 2014). Our study collected data related to cerebral oxygen saturation and analysed the baseline, intraoperative minimum value, and maximum variability of rSO_2_. It was found that the maximum variability of rSO_2_ during left cerebral operation was associated with POCD, and the incidence of DNR decreased while the maximum variability decreased. CO_2_ partial pressure, cerebral vasodilation, and cerebral perfusion (CBF) were elevated in patients undergoing laparoscopy with CO_2_ under certain pressure fillings of the abdominal cavity (Kim et al. 2016). However, further study revealed that the gap between the oxygen content of cerebral arteries and veins decreased along with a parallel increase in CBF, so the cause of the CBF increase is the lack of oxygenation of brain cells, i.e. hypoxia of brain tissue. Basic research has confirmed that cerebral ischaemia and hypoxia can cause postoperative cognitive dysfunction through a variety of mechanisms. Inflammatory factors, such as β-amyloid protein, il-1β/TNF-α, lactic acid, and highly active free radicals, act on DNA chains and organelles and even directly induce neuronal apoptosis (Hong et al. 2008; Kim et al. 2016; Sriram and O’Callaghan 2007; Zhu et al. 2006). In addition, studies have shown that there was no direct correlation between rSO_2_ and oxygenation, as all rSO_2_ drop events occur when SpO_2_ is greater than 98% (Green 2007). Therefore, intraoperative real-time monitoring of rSO_2_ plays an important role in preventing postoperative cognitive delay.

Currently, there are many studies on the risk factors for DNR, but few reports exist on how to observe these factors visually in the clinic. Although the prediction model and the nomogram constructed in this study had moderately low specificity, all the prediction indices are easy to obtain in clinical practice, and some indices (the minimum cerebral oxygen level) can be effectively interfered with, which can still assist clinicians in individually assessing the risk of DNR in patients undergoing laparoscopic resection for colorectal cancer and in adopting accurate prevention strategies. In addition, this research carried out internal and external validations, along with an efficiency appraisal of the predictive model. In the modelling group, the area under the ROC curve (AUC) obtained by the bootstrap method in internal validation were 0.751, and the AUC in the external validation were 0.694, which demonstrated a good predictive ability of the model.

## Limitation

This study was a single-centre study, so there may be some selection bias and the number of external validation cases included was limited. Large samples of multicentre data are required to further enhance the predictive value of the model.

## Conclusions

This study is the first to establish and validate a predictive model of delayed neurocognitive recovery associated with cerebral oxygen saturation monitoring. The risk prediction model of DNR related to cerebral oxygen saturation monitoring shows good predictive performance and clinical value, providing a basis for postoperative DNR prevention.

## Data Availability

The datasets used and analysed during the current study are available from the corresponding author on reasonable request.

## Abbreviations

DNR: Delayed neurocognitive recovery
POCD: Postoperative cognitive dysfunction
ASA: American Association of Anaesthesiologists
TIA: Transient ischemia attack
BMI: Body mass index
MMSE: Mini-mental State Examination
STROBE: Strengthening the Reporting of Observational Studies in Epidemiology
IAP: Intra-abdominal pressure
rSO_2__v_: Maximum variability of cerebral oxygen
CAD: Coronary artery disease
IQR: Interquartile range
CBF: Cerebral perfusion
